# Demographic-Adjusted Normative Equations for the Oculo-Cognitive Addition Test (OCAT): A Foundation for Individualized Oculo-Cognitive Assessment

**DOI:** 10.3390/jemr19050113

**Published:** 2026-10-02

**Authors:** Gaurav N. Pradhan, Sarah E. Kingsbury, Jan Stepanek, Michael J. Cevette, Richard J. Caselli

**Affiliations:** 1 Aerospace Medicine and Vestibular Research Laboratory, Mayo Clinic Arizona, Scottsdale, AZ 85259, USA; kingsbury.sarah@mayo.edu (S.E.K.); stepanek.jan@mayo.edu (J.S.); mcevette@mayo.edu (M.J.C.); 2 Department of Otolaryngology-Head and Neck Surgery, Division of Audiology, Mayo Clinic Arizona, Scottsdale, AZ 85259, USA; 3 Department of Biomedical Informatics, Mayo Clinic Arizona, Scottsdale, AZ 85259, USA; 4 Aerospace Medicine Program, Department of Internal Medicine, Mayo Clinic Arizona, Scottsdale, AZ 85259, USA; 5 Department of Neurology, Mayo Clinic Arizona, Scottsdale, AZ 85259, USA; caselli.richard@mayo.edu

**Keywords:** cognitive performance, cognitive screening, neurocognitive testing, oculomotor, eye movements, fixation, saccade, Oculo-Cognitive Addition Test

## Abstract

Background: Many neurological and cognitive disorders have early oculomotor hallmarks. The Oculo-Cognitive Addition Test (OCAT) is a quick, objective screening tool that measures eye movement and time-based biomarkers of cognitive processing during simple mental addition tasks in under two minutes. Normative values for age, sex, education level, and apolipoprotein E Type 4 (APOE ε4) carrier status must be established so cognitively normal populations are not misdiagnosed. Methods: A total of 382 participants completed OCAT, with 350 participants making up the normative cohort from which raw gaze data were processed to derive fixations, saccades, blinks, pupillary dynamics, and time-based features. Separate multiple linear regression models were fitted for each feature with age, sex, education, and APOE ε4 carrier status as predictors to establish demographic-adjusted normative equations. Results: Processing time, fixation time, fixation size and fixation area and their variability increased progressively from the first to the third number within each addition sequence, consistent with increasing cognitive workload. Demographic-adjusted multiple linear regression models generated normative equations for individual OCAT features based on age, sex, education, and APOE ε4 carrier status. These models were used to derive individualized expected values, 95% reference intervals, and z-scores. Conclusions: These demographic-adjusted normative equations enable individualized interpretation of OCAT performance and are necessary steps towards clinical implementation.

## 1. Introduction

The eyes, and particularly eye movements, provide a unique window into cognitive processing and neurological function. Effects of early-stage cognitive decline due to Alzheimer’s disease [1,2,3,4], Parkinson’s disease [5,6], and multiple dementias [7,8] can be reflected in subclinical changes to eye movement patterns. Eye movement abnormalities can also reveal early signs of diseases that affect motor abilities, like multiple sclerosis (MS) [9] or amyotrophic lateral sclerosis (ALS) [10], reflecting the effects of demyelinating lesions and motor neuron deterioration on optic nerves, cerebellar pathways, and the brainstem. Oculomotor abnormalities are frequently present in early stages of disease and often precede other noticeable vestibular or motor symptoms [11,12]. Furthermore, some reversible causes of cognitive impairment, like concussion [13] or metabolic conditions [14], can also disrupt normal oculomotor control. Together, these findings highlight the potential of eye movements as sensitive biomarkers of neurological dysfunction.

Advances in eye-tracking technology have enabled objective, non-invasive quantification of oculomotor behavior, making eye movement analysis an increasingly attractive tool for neurological screening. However, eye movements recorded during simple visual tasks may not impose sufficient cognitive load to reveal subclinical oculomotor signs of disease that emerge when multiple cognitive domains are simultaneously engaged. In contrast, eye movements recorded during cognitively demanding tasks can provide insights into higher cortical processes, like working memory, executive function, and processing speed [15]. These can reveal subclinical signs of disease or impairment, including abnormalities in smooth pursuit, saccadic intrusions, fixation instability, and nystagmus, providing greater sensitivity for detecting early neurological impairment to facilitate timely intervention and potentially delay disease progression.

Current diagnostic pathways for cognitive impairment present significant barriers. Gold-standard comprehensive neuropsychological evaluations typically require 4 to 6 h of specialized testing administered by trained personnel, limiting accessibility for many patients [16]. Moreover, early signs of these diseases are often overlooked in primary care settings [17,18,19], delaying diagnosis and reducing opportunities for intervention.

These limitations highlight the need for a rapid, objective screening tool capable of identifying individuals at elevated risk for cognitive impairment and neurological diseases. Such a tool could substantially improve diagnostic efficiency and optimize the use of limited neurology resources. The Oculo-Cognitive Addition Test (OCAT) [20] is a rapid digital assessment that combines tracking eye movements during cognitively demanding mental addition tasks using single-digit numbers, completed approximately under one minute [21,22]. OCAT leverages the well-established relationship between eye movement behavior and cognitive function [23,24]. Growing evidence demonstrates that abnormalities in fixation patterns, saccadic dynamics, and other oculometric measures [13,21,25] emerge during the earliest stages of cognitive decline and may reflect dysfunction in neural networks [22]. OCAT systematically imposes a progressively increasing cognitive workload, as participants sequentially add three single-digit numbers presented at 24 varying screen locations across 12 addition sequences. This simultaneously engages attention, working memory, executive function, and processing speed while eliciting horizontal, vertical, and diagonal eye movements. These movements are captured as potential oculomotor biomarkers of cognitive and motor impairments. An integrated eye-tracking system captures detailed oculometric measures, including scan-paths and temporal metrics reflecting voluntary and involuntary cognitive processes.

Before OCAT can be used to determine abnormal performance, however, normative values for age, sex, and education level must be determined. Cognitive change happens across the lifespan, with some impairment occurring in unpredictable, pathological ways, and some decline following natural, expected patterns. Since normal aging has effects on oculometric biomarkers [26,27], it is important to measure how these differences across older adulthood manifest. Additionally, there could be interaction effects between aging and eye movements that need to be labelled so they are not misinterpreted as signs of disease in cognitively normal aging participants. Impairment should be diagnosed as a patient’s performance relative to age-adjusted norms [28].

Aging effects are not the only factors that must be accounted for when determining normative values for OCAT’s eye movement and time features. Contemporary researchers argue that demographic corrections to popular neuropsychological tests should be completed not only for age, but also for sex and education level [29]. In the case of the Auditory Verbal Learning Task (AVLT), if biological sex was ignored, older men would be over-diagnosed with cognitive decline and older women would be underdiagnosed by this test [29]. Alternatively, some demographics emanate from life experience and lifestyle choices, like years of education. While there is no conclusive evidence that increased years of education are protective against cognitive decline, they have been shown to modify the relationship of Alzheimer’s-related plaques to level of cognitive function in older adults [30]. Additionally, years of education have been shown to affect neuropsychological test scores, which require scaling to correspond to magnetic resonance imaging (MRI)-based measures of dementia, education level notwithstanding [31]. It is crucial that all test-takers are assessed on an even field to differentiate between true neurological disease and normal cognitive processing differences resulting from age, sex, and education.

The objective of this work is to generate normative scores for eye movement and time-related OCAT features that scale for the influences of age, sex, education level, and apolipoprotein E Type 4 (APOE ε4) carrier status. Carrying alleles of APOE ε4 is one of the strongest known genetic risk factors for Alzheimer’s disease, with increasing risk for disease and early onset based on the number of alleles inherited [32]. Importantly, having a genetic predisposition does not mean that a patient necessarily has the disease nor that they show symptoms. Since all cohort members received genetic testing that determined their APOE allele types, the research team deemed it scientifically interesting to assess if APOE ε4 carrier status in cognitively normal individuals had meaningful effects on OCAT features.

In this study, eye movement and time-based features were extracted from the raw gaze data collected during OCAT assessments that were performed at the onset of full neuropsychological testing appointments in the Department of Neurology [33]. All participants in the current cohort have been labelled as cognitively normal, based on their complete testing, including the Dementia Rating Scale (DRS) and AVLT, as well as imaging interpreted by a neurologist. It was hypothesized that increasing age would be associated with slower OCAT-derived oculomotor and cognitive performance, reflected by longer completion times, increased saccadic latencies, longer fixation durations, and larger fixation sizes and areas. Additionally, higher educational attainment was expected to be associated with more efficient task performance, reflected in shorter OCAT completion times and faster processing during the sequential addition task (i.e., Number 1 + Number 2, and then (Number 1 + Number 2) + Number 3). The future aim of this work is to develop a fast, accurate screener for neurocognitive diseases, which requires adjusted models to identify normal results.

## 2. Materials and Methods

### 2.1. Participants

A total of 382 cognitively normal participants from the longitudinal study of the Arizona Apolipoprotein E (APOE) Cohort [33] completed the OCAT as part of their evaluation at the Department of Neurology, Mayo Clinic Arizona. Cognitive normality was determined based on neuropsychological assessment and independent evaluation by a study neurologist. The study protocol was approved by the Mayo Clinic Institutional Review Board (IRB), and all participants provided informed consent. Participants were required to have normal vision (including normal vision with correction). OCAT was conducted prior to all neuropsychological testing to neutralize fatigue effects. Data from 32 participants were excluded because their eye-tracking recordings during OCAT testing did not meet the predefined minimum “tracking ratio” threshold of 80% (due to signal loss, poor calibration, unstable gaze, etc.). Therefore, the final normative cohort consisted of 350 participants. Table 1 summarizes the demographic and clinical characteristics of the normative cohort, including age, sex, years of education, and APOE ε4 carrier status. These variables were incorporated into the regression models to establish demographic-adjusted normative equations for individual OCAT features based on eye movements (fixations, saccades, blinks and pupillary dynamics) and cognitive performance.

### 2.2. Equipment

The OCAT was conducted in a quiet, well-lit, climate-controlled room at Mayo Clinic Arizona. The OCAT software (version 1.0) was installed on and administered using a 14” EyeOn Elite Windows 10 pro tablet (EyeTech Digital Systems, Tempe, AZ, USA). This built-for-purpose tablet was equipped with an integrated eye tracking device (8 MP Eye Gaze Camera, EyeTech Digital Systems, Tempe, AZ, USA) for continuous gaze recording during the OCAT. The tablet screen had a resolution of 1920 × 1080 pixels, and raw eye-tracking data were acquired at a sampling rate of 120 Hz. Participants were seated approximately 60 cm from the tablet, the recommended reading range distance, which was verified during the calibration procedure. Before testing, a five-point calibration procedure was performed to optimize eye-tracking accuracy and recording quality. Calibration quality was verified after one calibration session, and recalibration was performed when necessary to achieve an error of less than 0.5 degrees. A guided preview was provided before data acquisition to minimize task novelty effects, followed by a practice session in which participants completed one full OCAT trial. This practice trial familiarized participants with the task and reduced learning-related variability. The subsequent OCAT trial served as the formal test, and only data from this trial were included in the analyses.

### 2.3. OCAT Design and Process

The Oculo-Cognitive Addition Test (OCAT) [20] is a digital assessment lasting approximately one minute that measures eye movements during progressively demanding mental addition tasks [21,22]. Participants sequentially add three single-digit numbers presented at varying spatial locations while their eye movements are continuously recorded. Across 12 addition sequences and 24 screen locations, OCAT elicits horizontal, vertical, and diagonal eye movements during systematically progressing cognitive workload. The integrated eye-tracking system quantifies detailed oculometric measures, including fixation patterns, saccadic dynamics, scan-paths and temporal metrics reflecting voluntary and involuntary cognitive processes [13,21,25]. Rather than measuring cognitive performance or eye movements in isolation, OCAT captures the oculometric features associated with the cognitive effort required to perform mental computation.

OCAT’s architecture is designed to simultaneously impose a graded cognitive workload with multidirectional oculomotor demands. The test consists of completing, as rapidly as possible, 12 sequences of summing three consecutive single-digit numbers shown separately on three consecutive blank screens in seemingly random positions (Figure 1). The numbers are presented across the full active display area of a 14-inch tablet, measuring 31.0 × 17.4 cm with a 16:9 aspect ratio. As shown in Figure 1, each single-digit number is displayed in 24-point bold font with black text on a light-green background for the first two numbers and a light-red background for the final number in each sequence; the background is sized to fit the numeral displayed rather than using a pre-defined fixed-size bounding box. Within each sequence, cognitive demand progressively increases from low to medium to high workload. Low cognitive workload occurs when the participant locates, reads, and calls out the first number (e.g., 5). Medium cognitive workload occurs when the participant is required to locate the second number, recall the first, mentally add the two, and call out their sum (e.g., 5 + 7 = 12). High cognitive workload occurs when the participant locates the third number, retains the preceding sum in working memory, adds the third number, and calls out the final sum (e.g., 12 + 6 = 18). Each sequence uses a different combination of numbers and spatial locations, repeatedly cycling the participant through progressively increasing cognitive demands. Participants were instructed to maintain fixation on the currently displayed number until responding and then visually locate the subsequently presented number.

Although each number’s location appears unpredictable to the participant, the spatial sequence follows an underlying “infinity loop” structure comprising 24 symmetrical positions (Figure 2 Left). The 12 addition sequences are systematically distributed to elicit horizontal, vertical, and diagonal eye movements with each three-number sequence directing gaze predominantly along a defined movement orientation. Hence, while target locations appear random and cannot be anticipated by the participants, their underlying spatial organization is controlled. This architecture requires active visual search and enables systematic quantification of oculomotor responses across multiple directions while cognitive workload increases within each sequence, from low to medium to high.

Throughout the task, an integrated eye-tracking system continuously records the raw eye movement scan-paths (Figure 2 Right), enabling the extraction of both involuntary and voluntary oculometric features associated with cognitive effort. The red clusters are classified as fixations, which is the ability of a person to sustain visual focus on a target. This measure is representative of the involuntary effects of cognitive processing and effort. Abnormalities in fixation (i.e., presence of intrusive saccades during a fixation task) have long been reported as biomarkers of AD [1,4]. The blue lines represent saccadic movements which are generated when the subject locates a new number between screens. Saccades are rapid, conjugate eye movements that can occur in response to voluntary focus on an object of interest (e.g., during tracking or reading). OCAT also quantifies saccade latency, which is the time elapsed from the appearance of a visual target to the initiation of saccadic movement and subsequent visual fixation on the target. In addition, several pupil-based and blink-related features are extracted to capture physiological responses potentially indicative of cognitive load.

### 2.4. OCAT Performance Features and Data Processing

OCAT-derived features were categorized into two domains: time-related and eye movement-related features. The eye movement-related features are further categorized into (1) fixation features, (2) saccadic features, (3) blink features, and (4) pupillary features (Table 2).

Time-related features quantified both overall task performance and the speed and consistency of cognitive processing during each stage of the addition task. These included overall test completion time (Total Test Time) and the mean and standard deviation (SD) of the time required to process the first, second, and third numbers across the 12 addition sequences that comprise a single OCAT assessment (Mean Time and SD for Number 1, 2, 3). The processing time for each number was calculated independently of the gaze data as the time elapsed between presentation of the number and the participant’s subsequent spacebar press to advance to the next number, thereby providing separate processing times for the first, second, and third numbers within each addition sequence. The time associated with Number 1 primarily reflects sequence initiation and visual acquisition of the first number before any arithmetic operation is performed, whereas the times for Numbers 2 and 3 incorporate the additional cognitive processing required for sequential mental addition. The corresponding SD measures quantify within-assessment variability in processing time across the 12 addition sequences and provide an index of the consistency of cognitive performance.

The raw gaze data were pre-processed before eye-movement related features were extracted. Artifacts were removed with filtering and interpolation-based spike correction compared to neighboring valid samples. This was done to eliminate outliers or physiologically implausible gaze samples that could skew the data. Next, fixations and saccades were classified using the classification approach described previously [13]. A saccadic threshold of 1° of visual angle, corresponding to approximately 10.5 mm of sample-to-sample displacement on the display at the 60 cm viewing distance, was used to distinguish saccadic transitions from within-fixation gaze movements. Gaze movements exceeding this threshold were classified as saccadic movements, whereas movements within the threshold were considered part of a fixation. Because saccade identification was based on the spatial distance between consecutive gaze samples relative to this threshold, portions of the eye movement near the saccade boundaries may not be classified as part of the saccade. Thus, the duration of detected saccades may be underestimated, with a corresponding overestimation of the classified fixation duration between successive saccades.

Fixation behavior was characterized using three complementary measures: fixation time (also commonly known as fixation duration), fixation size and fixation area. Fixation time represents the temporal characteristic of fixations and is defined as the time elapsed between the beginning and end of a fixation. Fixation size characterizes the cumulative spatial movement of gaze within a fixation and is defined as the total spatial scan path length obtained by summing the point-to-point distances between consecutive gaze samples within that fixation. In contrast, fixation area characterizes the spatial dispersion of the fixation and is calculated as the area of the convex hull encompassing all sampled gaze data points within that fixation. Thus, accordingly, fixation size quantifies how much distance the gaze travels within a fixation, whereas fixation area quantifies how broadly those gaze points are distributed in space [13]. When multiple classified fixations were associated with the processing of a given number, fixations with spatially overlapping convex hulls were merged and treated as a single fixation. For multiple non-overlapping fixations, fixation time, size and area were summed to obtain the corresponding fixation measures for that number.

For each trial of OCAT, mean fixation time, mean fixation size, and mean fixation area were calculated across fixations across 12 sequences during low, medium, and high cognitive workload. To additionally characterize intra-trial variability, the standard deviations of fixation time, size, and area within the 12 sequences during low, medium, and high cognitive workload were calculated and termed fluctuation in fixation time, fluctuation in fixation size, and fluctuation in fixation area, respectively.

Within each three-number sequence of the OCAT assessment, the intended saccadic eye movements were arranged in a horizontal, vertical, or diagonal direction, with four sequences corresponding to each direction. Saccades are rapid, ballistic, conjugate eye movements that change the line of sight between fixation locations [34] and can occur in response to voluntary focus on an object of interest (e.g., tracking [23,35] or reading [36]). Saccadic performance was characterized using measures of latency, velocity, and path lengths. Saccade latency is the time elapsed from the appearance of a visual target to the initiation of saccadic movement [23]. In OCAT, mean saccade latency was operationally defined as the time elapsed between stimulus onset (i.e., number appearing on the screen), and initiation of the subsequent saccadic eye movement exceeding the 1° saccadic threshold. Small fixational saccades (≤1°) were therefore excluded from this measure. However, because very short-latency saccades (>1°), were not independently classified as anticipatory versus visually driven, this measure represents an operational post-target saccade latency rather than exclusive measure of visually driven primary-saccade latency. Saccadic velocity is the angular speed of the eye during a saccadic movement [37,38]. Mean saccadic velocity (deg/s) was calculated separately for the horizontal, vertical, and diagonal target-transition directions.

The total saccadic path length was calculated to represent the cumulative angular distance traveled by the eyes during all detected saccades occurring within the four sequences associated with all three directions, regardless of the direction of the individual saccades. In contrast, we also measured direction-specific saccadic path length that represents the cumulative angular distance traveled only by saccades classified as horizontal, vertical, or diagonal during the corresponding four sequences. Thus, the former quantifies overall eye movement required to complete the task, whereas the latter quantifies eye movements specifically aligned with the intended target transition direction.

Finally, blink and pupil-based features were extracted to characterize physiological responses associated with oculomotor and cognitive load during OCAT. A blink was operationally defined as a continuous period in which valid gaze coordinates were absent for both eyes, represented by (−1, −1) coordinates in consecutive samples of raw eye movement data. At 120 Hz sampling rate (~8.3 ms/sample), periods of bilateral missing gaze data lasting between 100 ms and 500 ms were classified as blinks, consistent with physiologically plausible blink durations. Blink rate was calculated as the total number of detected blink events per minute of OCAT task engagement, providing a measure of blink frequency. Median blink duration representing the typical length of the blink was calculated as the median duration of detected blinks during OCAT.

Pupillary dynamics were characterized using the standard deviation of pupil size and the coefficient of variation in pupil size. The standard deviation of pupil size was calculated from continuous pupil diameter measurements during OCAT, reflecting intra-individual variability in pupil dynamics and potential sensitivity to task demands. To quantify cognitive stress responses, the coefficient of variation in pupil size was calculated as the ratio between the standard deviation of the pupil diameter and the mean pupil diameter during OCAT.

### 2.5. Data Analysis

Pre-processing of the raw gaze data and extraction of eye movement-related features were performed using MATLAB R2024a (Mathworks, Natick, MA, USA). Statistical analyses were conducted in Python (version 3.12.7) using the statsmodels library (version 0.14.6). Separate multiple linear regression models were fitted for each OCAT feature with age, sex, years of education, and APOE ε4 carrier status as prespecified predictors to establish demographic-adjusted normative equations. Model performance was summarized using adjusted R^2^ and the root mean squared error (RMSE). For each multiple linear regression model, residual skewness was evaluated. An absolute residual skewness value >1 was used as a practical criterion for considering transformation. In all models meeting this criterion, residual skewness was positive (>1); therefore, the corresponding OCAT-derived feature outcome was natural log-transformed (ln) to reduce positive skewness, and the regression model was refitted. The residual distribution of each refitted model was subsequently re-evaluated. For interpretability, RMSE for models fitted to ln-transformed outcomes was reported in the original units by back-transforming the predictions and calculating the RMSE using the original observed values. This original-scale RMSE was used for reporting purposes only; z-score and reference limit calculation used the residual RMSE from the model on the ln scale. Model assumptions were evaluated by examining residual distributions, residual skewness and kurtosis, residual-versus-fitted plots, the Breusch–Pagan test for homoscedasticity, and variance inflation factors to assess multicollinearity. For models demonstrating evidence of heteroscedasticity, HC3 heteroscedasticity-consistent standard errors were used to obtain robust confidence intervals and *p*-values. Regression equations were subsequently used to derive adjusted normative reference values and reference intervals according to age, sex, education, and APOE ε4 carrier status.

## 3. Results

Of the 382 cognitively normal participants who completed OCAT testing, 350 had a tracking ratio greater than 80%, the pre-defined eye-tracking data quality criterion. No clear age-related pattern was observed among the excluded participants because of inadequate eye-tracking quality. However, ocular conditions and technical factors affecting reliable eye detection may contribute to reduced tracking quality and warrant further evaluation, particularly when extending OCAT to older and clinically involved populations. These 350 participants comprised the normative cohort used to characterize the distribution of OCAT-derived oculomotor and cognitive performance features, detailed below. Following these results are the demographic-adjusted normative regression equations.

### 3.1. Descriptive Statistics of OCAT Features

Descriptive statistics for the cognitive and oculomotor performance features derived from the OCAT for the 350 cognitively normal participants are presented in Table 2. The summarized features encompass five domains: time-related performance, fixations, saccades, blinks, and pupillary dynamics. For each feature, the mean, standard deviation, median, 25th percentile (P25), 75th percentile (P75), minimum, and maximum values are reported to characterize the distribution of normative performance within the study cohort. These descriptive statistics provide the basis for the development of demographic-adjusted normative equations and reference values.

The mean total OCAT completion time was 66.5 ± 14.1 s, with a median of 64.9 s (interquartile range: 56.7–74.7 s). The mean processing times increased progressively across the three sequential numbers within each addition sequence, averaging 1.67 s for Number 1 (low cognitive workload), 1.71 s for Number 2 (medium cognitive workload), and 2.16 s for Number 3 (high cognitive workload). The standard deviation of the processing time for each number, calculated across the 12 sequences within a single OCAT assessment, also increased from Number 1 to Number 3.

Fixation-related measures also demonstrated longer fixation durations and greater variability as participants progressed through the addition sequence. Mean fixation time increased from 1233 ms for Number 1 to 1557 ms for Number 3, accompanied by corresponding increases in fixation time fluctuation. Similarly, fixation size and fixation area increased across the three processing stages, consistent with increasing cognitive demands during sequential calculation.

The median saccade latency was approximately 0.52 s, while median saccadic velocities differed according to movement direction, averaging 302 deg/s for diagonal saccades, 325 deg/s for horizontal saccades, and 279 deg/s for vertical saccades. Total saccadic path lengths also varied by movement direction.

Blink measures demonstrated considerable inter-subject variability, with a mean blink rate of 17.9 blinks/min, and a mean blink duration of 195 ms. Pupillary dynamic measures were comparatively stable. Across the majority of OCAT features, the proximity of mean and median values suggested approximately symmetrical distributions, although several fluctuation measures exhibited broader ranges and greater inter-subject variability.

### 3.2. Demographic-Adjusted Normative Regression Models

Demographic-adjusted normative equations were developed for each of the 42 OCAT-derived oculomotor and cognitive features using multiple linear regression models with age, sex, education, and APOE ε4 carrier status as prespecified predictors. The resulting regression coefficients, intercepts, adjusted R^2^, and root mean squared error (RMSE) for each feature are summarized in Table 3. For instance, these equations provide the basis for estimating expected value of OCAT feature and deriving individualized normative reference values adjusted for demographic and genetic characteristics as follows:

OCAT feature = *β*0 + *β*1(*age value*) + *β*2(*sex value*) + *β*3(*education value*) +*β*4(*APOE value*) (1)

where sex and APOE ε4 carrier status values are coded as binary variables (female = 0, male = 1; and ε4 non-carrier = 0; ε4 carrier = 1). For ln-transformed outcomes, the RMSE reported in Table 3 was calculated in the original units using back-transformed model predictions and observed values.

For time-related cognitive performance features, age was a significant predictor for Mean Time for Number 1 and Std. Dev. Time for Number 1. Age was again a significant predictor for most fixation features related to Number 1 and Fluctuation in Fixation Size for Number 3. Similarly, within saccadic features, Mean Saccade Latency, Total Saccadic Path Length during Diagonal sequences, Total Diagonal Saccadic Path Length, and Median Diagonal Saccadic Velocity observed a significant association with age. Sex was a significant predictor for Blink Rate, and Fluctuation in Blink Duration. The negative regression coefficients indicate that male category (1) had lower predicted values than female category as the reference (0). In addition, withing pupillary features, Mean Pupil Diameter and Fluctuation in Pupil Diameter observed a significant association with age. Overall, there were limited significant findings for sex and education, with few significant findings for APOE ε4 carrier status. APOE ε4 carrier status was a significant predictor for Std. Dev. of Time for Number 2 and two blink variables, Mean Blink Duration and Fluctuation in Blink Duration.

Model diagnostics are summarized in Table 4. Overall, the multiple regression models demonstrated acceptable performance across the OCAT features. Residual distributions evaluated using residual skewness and kurtosis were close to values expected for approximately normal residuals across all OCAT-derived features. Evidence of heteroscedasticity using the Breusch–Pagan test was observed for only nine of the 42 features (*p* < 0.05). As mentioned before, for these features, HC3 heteroscedasticity-consistent standard errors were applied to obtain robust confidence intervals. Multicollinearity among the predictors was negligible, with all variance inflation factors close to 1.0 and a maximum VIF of 1.02. Collectively, these findings support the suitability of the regression models for developing demographic-adjusted normative ranges for OCAT-derived eye movement and cognitive performance measures.

### 3.3. Implementing Regression Models

Examples of demographic-adjusted normative reference values derived from the above regression equations are presented in Table 5 and Table 6 for two representative OCAT features: Fluctuation in Fixation Time for Number 1 and Mean Saccade Latency. The reference tables demonstrate how expected OCAT performance and corresponding 95% reference levels can be individualized to age, sex, and APOE ε4 carrier status, with education held constant at 16 years. For the fluctuation in fixation time for Number 1, expected values increased progressively with age, with corresponding upward shifts in the lower and upper reference limits. Female participants exhibited slightly higher-than-expected values compared to males with the same age, education level, and APOE status.

Similarly, expected saccade latency increased with age across both sexes and APOE groups. The corresponding 95% reference levels shifted upwards with increasing age. Differences associated with sex and APOE ε4 carrier status were small relative to the age-related difference.

For clinical interpretation, an individual participant’s standardized score for the OCAT feature, say *f*, can be calculated by subtracting corresponding demographic-adjusted expected value from the observed OCAT feature value of f and dividing by the corresponding model RMSE for f from Table 3 as follows:

$$z_{f}=\;\frac{{\mathrm{Observed}\;\mathrm{Value}}_{f}-{\mathrm{Expected}\;\mathrm{Value}}_{f}}{{\mathrm{RMSE}}_{f}}$$

For models fitted after log-transformation of the outcome, the z-score is calculated on the log scale as follows:

$$z_{f}=\;\frac{{\ln(\mathrm{Observed}\;\mathrm{Value}}_{f})-{\ln(\mathrm{Expected}\;\mathrm{Value}}_{f})}{{\mathrm{RMSE}}_{f}(\ln)}$$

A z-score of 0 indicates performance equal to the expected value of an individual with the same age, sex, education, and APOE ε4 carrier status. Scores further away from 0 indicate increasing deviation from the normative expectation. The 95% reference interval was defined as the model predicted value ± 1.96 * RMSE. For ln-transformed outcomes, the limits were calculated on the ln scale and subsequently back-transformed to the original units.

## 4. Discussion

### 4.1. Descriptive Findings

On average, participants in the normative cohort completed the OCAT in approximately one minute (mean = 66.53 s). Mean processing time increased progressively from Number 1 to Number 3 (1.67–2.16 s), consistent with the increasing cognitive demand imposed by sequential mental addition. Similarly, the within-assessment variability quantified by the standard deviation of processing time for Numbers 1–3 increased from 0.39 to 0.76 s. This pattern suggests that as participants progressed through each addition sequence, the increasing cognitive workload not only prolonged processing time but also resulted in greater variability in cognitive processing. This observation likely reflects greater fluctuation in attention, working memory, and executive control as the mental computation became progressively more demanding.

### 4.2. Effects of Age on OCAT Feature Performance

Age was the demographic factor most associated with OCAT feature performance; there were some limited associations with sex and education level. Potential non-linear age effects were examined using quadratic and spline terms and did not meaningfully improve model fit compared with linear age models. Given the relatively smaller representations at the extremes of age distribution, potential non-linear age effects warrant further evaluation in larger and more age-diverse cohorts. Although it was hypothesized that aging primarily affects overall OCAT task completion time and performance during high cognitive workload, age-related effects were most evident in the first step of every new addition sequence (i.e., during Number 1), which represents the lowest cognitive workload. Specifically, increasing age was associated with longer mean processing time for Number 1, which corresponds to the interval from presentation of the first number of a new sequence to the participant’s subsequent spacebar press after reading the number aloud. Although Number 1 in an OCAT sequence requires participants to locate and read the number, it also requires them to disengage from the preceding sequence and initiate the new one. This transition places demands on task switching [39,40,41], a component of cognitive flexibility associated with executive function [42]. Successful performance requires suppressing the previous running total, remembering the task instructions in working memory, and shifting from completing one sequence to initiating the next by reading a number and not adding. Consequently, the cognitive demands during Number 1 extend beyond simple visual recognition and reading aloud, incorporating executive processes involved in switching between mental sets.

Age-related declines in task-switching ability and cognitive flexibility are well-documented and are associated with slower and less efficient executive control [40] in older adults. These typical changes in cognitively normal older adults may explain why Number 1 performance was more sensitive to aging effects than Numbers 2 and 3, despite involving the lowest arithmetic workload. Our findings suggest that the transition into a new addition sequence, which establishes the cognitive set for the subsequent arithmetic computations, may be especially sensitive to age-related changes in executive function.

Interestingly, age was associated with more measures of within-assessment variability (fluctuation measures) than with mean performance measures. Within-assessment variability, or fluctuation, reflects the consistency of an individual’s performance across 12 addition sequences and was quantified as the standard deviation of repeated observations during a single OCAT assessment. For example, increasing age was associated with greater variability in the processing time for Number 1, indicating older adults exhibited less consistent performance rather than simply slower performance. In other words, their processing times fluctuated more across all sequences in the assessment, whereas younger counterparts performed more consistently. This aligns with existing research that shows older adults (54–94 years) demonstrate increased within-assessment inconsistency in reaction times than younger adults (17–36 years), even after controlling for mean reaction times [43,44]. Increased within-assessment variability is a potential marker of reduced stability in cognitive processing and may reflect age-related declines in executive control and attentional regulation.

### 4.3. Measures of Variance

Most regression models demonstrated adjusted R^2^ values below 0.05 (Table 3), indicating that age, sex, education, and APOE ε4 carrier status collectively explained a comparatively small (<5%) variance in OCAT feature performance amongst cognitively normal adults. While these findings suggest that OCAT-derived features are relatively stable across common demographic characteristics, it is also possible that substantial interindividual variability remains unexplained and may reflect factors not included in the models. External validation in more heterogeneous populations is needed to determine whether these findings persist across broader demographic and clinical populations. The minimal contribution of APOE ε4 carrier status is not unexpected, as all participants were cognitively normal and APOE ε4 carrier status primarily confers increased risk for future cognitive decline rather than measurable cognitive impairment in asymptomatic individuals. Its inclusion in normative adjustment could potentially reduce the sensitivity to subtle oculomotor or cognitive changes associated with preclinical changes. Therefore, its role may be more appropriate as an exploratory modifier until its role in clinical normative models is established through longitudinal and external validation. Although demographic adjustment remains appropriate for establishing individualized normative reference values for this test, the modest influence of these variables indicates that OCAT performance is less dependent on demographic factors compared to many conventional neuropsychological measures.

This finding contrasts with established neuropsychological tests that demonstrate strong influence by demographic predictors. In 2024, researchers conducting the Mayo Normative Studies in the landmark Mayo Clinic Study of Aging, found that age, sex, and education explained up to 42.5% of the variance in WAIS-R Digit Symbol performance, 39.4% in AVLT performance, and 32.1% in Part B of the Trail Making Test [45]. Compared with these traditional measures, the relatively small demographic effects observed for OCAT suggest that it provides a more demographically robust assessment of cognitive and oculomotor performance while still benefitting from demographic-adjusted normative equations.

### 4.4. Clinical Applications

The regression models developed in this study enable the calculation of demographic-adjusted expected values, 95% reference intervals, and standardized z-scores for each of the 42 OCAT-derived features. Table 5 and Table 6 illustrate this approach using Fluctuation in Fixation Duration for Number 1 and Mean Saccade Latency Time, demonstrating how normative values can be individualized according to age, sex, education level, and APOE genotype.

Integration of these equations into the OCAT software will allow clinicians to compare a patient’s observed performance with the expected value for an individual with the same demographic characteristics. Feature performance falling outside the demographic-adjusted reference intervals or exhibiting abnormal z-scores could flag domains requiring further clinical evaluation. However, these normative values are intended to support, rather than replace, clinical judgement. The significance of an abnormal OCAT feature depends on a clinical context, as eye movement characteristics such as fixation instability (large fixation area) or gaze variability (high saccadic length) may reflect neurological dysfunction in some settings but may also occur in cognitively normal individuals performing a high mental load visual task [2,46,47]. OCAT-derived measures encompass both oculometric features derived from eye movement and oculo-cognitive performance features that reflect the integration of oculomotor and cognitive functions. In particular, processing time measures incorporate visual search, arithmetic processing, working memory, executive control, verbal response, and motor response required to advance the task, thereby reflecting integrated functional performance rather than isolated oculomotor function.

The demographic-adjusted normative equations developed in this study provide a standardized framework for individualized interpretation of OCAT performance and represent an important step toward precision medicine in neurological screening. However, these equations are cohort-derived, and external validation in populations with broader educational, racial/ethnic, socioeconomic, and geographic representation is warranted before their broader application as clinical normative standards. Importantly, abnormal z-scores should not be interpreted as diagnostic evidence of cognitive impairment. Instead, they identify as deviations from expected normative performance that may warrant additional neurological assessment. Diagnostic thresholds and classification performance should be established in future studies involving individuals with clinically confirmed cognitive impairment. In addition, future validation should evaluate the test-retest reliability and normal intraindividual variability in OCAT-derived features to support interpretation of longitudinal changes in individual z-scores and their potential clinical applications.

## 5. Conclusions

This study establishes the first demographic-adjusted normative equations for the OCAT, providing expected values, 95% reference intervals, and standardized z-scores for 42 oculomotor and cognitive performance features in cognitively normal adults. Age was the demographic factor most consistently associated with performance on OCAT-derived features, particularly fluctuation measures, or within-assessment variability, whereas sex, education level, and APOE ε4 carrier status had comparatively modest effects. The relatively small influence of these demographic characteristics suggests that OCAT-derived features are relatively stable amongst cognitively normal individuals but still benefit from demographic-adjusted interpretations.

These normative equations provide the foundation for individualized clinical interpretation of OCAT performance and represent an essential step toward implementing OCAT into clinical neurology workflows as a rapid, objective neurological screening tool. Future studies should validate the diagnostic performance of these normative models in individuals with mild cognitive impairment, dementia, and other neurological disorders to establish disease-specific thresholds and determine their utility for early detection and clinical decision-making for early management of neurological disorders.

## 6. Patents

Gaurav N. Pradhan, Michael J. Cevette, and Jan Stepanek, Oculo-Cognitive Addition Testing, US Patent 11,869,386 B2, filed 1 December 2016, and issued 9 January 2024.

## Figures and Tables

**Figure 1:**
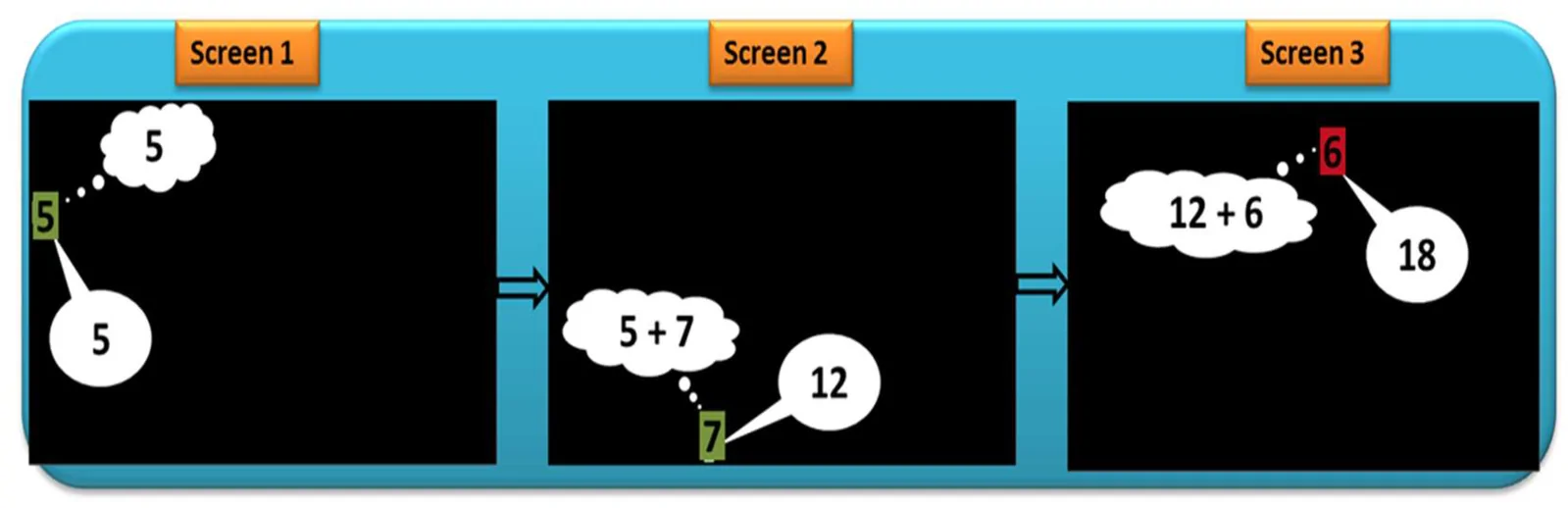
A visual representation of one trial of OCAT. 12 trials of OCAT are completed by each subject with a different set of three numbers in three different locations. Screens 1, 2, and 3 correspond to low, medium, and high cognitive workload, respectively. The cloud represents cognitive processing, and the oval represents the subject’s verbal response.

**Figure 2:**
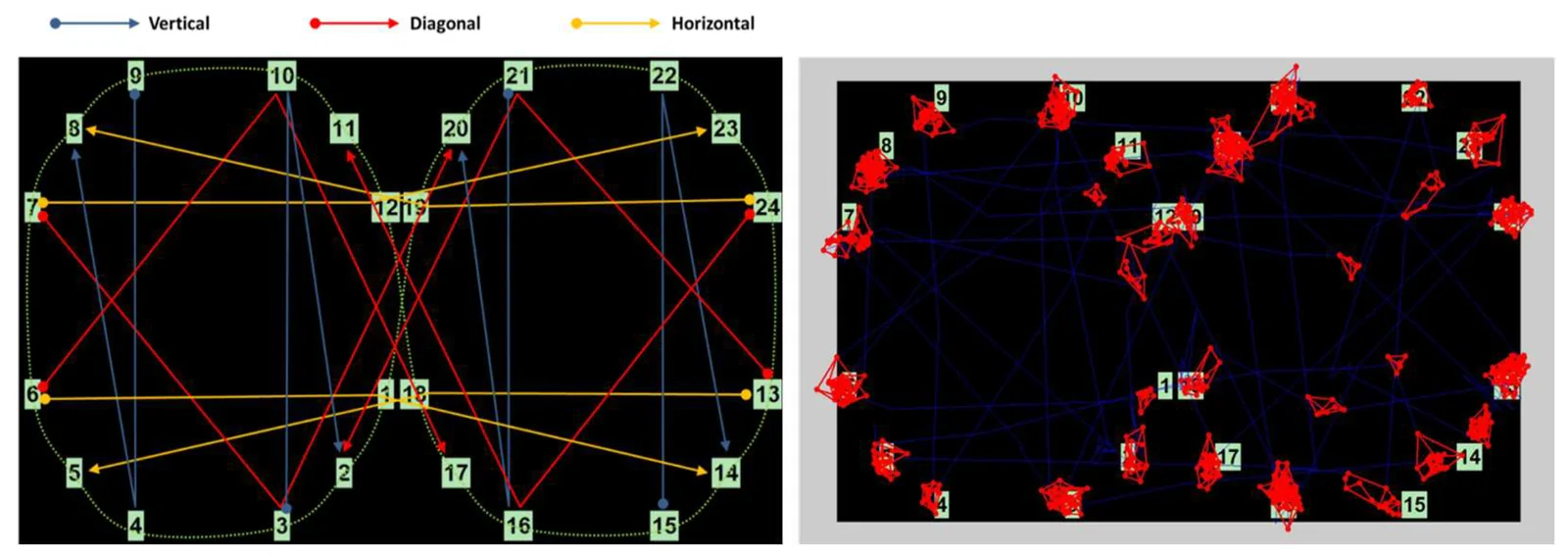
(**Left**) All 12 unique combinations of sequences in three categories: vertical (blue), diagonal (red), and horizontal (yellow). (**Right**) Eye tracking data during OCAT showing the fixations (red) and saccadic movement (blue).

**Table 1:** Demographic and clinical characteristics of the normative OCAT cohort (N = 350).

Characteristic	Overall (*N* = 350)
Age, years	65.4 ± 9.5
Median (IQR)	66 (59–72)
Range	31–91
Age groups, *n* (%)	
≤59 years	94 (26.9%)
60–69 years	126 (36%)
70–79 years	111 (31.7%)
≥80 years	19 (5.4%)
Female, *n* (%)	260 (74.3%)
Male, *n* (%)	90 (25.7%)
Education, years	16.6 ± 2.2
Median (IQR)	16 (16–18)
APOE ɛ4 carriers, *n* (%)	152 (43.4%)
APOE ɛ4 non-carriers, *n* (%)	198 (56.5%)
DRS	140.7 ± 2.7
AVLT	9.8 ± 3

**Table 2:** Descriptive statistics of OCAT features.

	OCAT Features	Mean	Std. Dev.	Median	P25	P75	Min	Max
Time-related Cognitive Performance Features	Total OCAT Test Time (s)	66.53	14.05	64.94	56.66	74.67	37.07	112.81
	Mean Time # 1 (s)	1.67	0.36	1.63	1.42	1.88	0.81	2.87
	Std. Dev. Time #1 (s)	0.39	0.25	0.32	0.21	0.53	0.07	1.84
	Mean Time # 2 (s)	1.71	0.4	1.64	1.43	1.92	0.84	3.24
	Std. Dev. Time # 2 (s)	0.44	0.28	0.37	0.24	0.56	0.08	2.34
	Mean Time # 3 (s)	2.16	0.53	2.1	1.77	2.44	1.09	4
	Std. Dev. Time # 3 (s)	0.76	0.47	0.63	0.43	1	0.15	2.64
Fixation Features	Mean Fixation Time # 1 (ms)	1233.08	347.05	1213.09	1007.7	1449.42	294.55	2363.79
	Fluctuation Fixation Time # 1 (ms)	365.02	168.56	324.39	244.58	446.88	88.86	975.97
	Mean Fixation Size # 1 (mm)	488.65	138.09	472.64	388.03	581.67	150.14	907.44
	Fluctuation Fixation Size # 1 (mm)	153.27	68.29	138.24	104.46	184.65	40.59	415.7
	Mean Fixation Area # 1 (mm^2^)	288.63	95.23	287.11	219.34	351.12	93.81	574.8
	Fluctuation Fixation Area # 1 (mm^2^)	113.84	49.2	107.68	77.9	138	28.63	290.63
	Mean Fixation Time # 2 (ms)	1244.03	354.75	1217.02	1013.33	1448.03	235.57	2529.07
	Fluctuation Fixation Time # 2 (ms)	359.48	161.14	330.73	243.14	444.91	96.54	1063.95
	Mean Fixation Size # 2 (mm)	482.04	146.11	467.7	369.27	570.1	133.62	969.79
	Fluctuation Fixation Size # 2 (mm)	144.97	64.54	129.46	96.27	179.19	29.84	371.56
	Mean Fixation Area # 2 (mm^2^)	262.55	87.48	256.14	200.63	313.69	78.41	508.73
	Fluctuation Fixation Area # 2 (mm^2^)	97.07	39.39	91.23	66.32	119.5	21.77	240.33
	Mean Fixation Time # 3 (ms)	1556.62	467.26	1544.32	1230.98	1821.49	510.77	3076.01
	Fluctuation Fixation Time # 3 (ms)	526.73	284.03	453.09	315.17	647.5	46.52	1690.16
	Mean Fixation Size # 3 (mm)	608.25	187.28	591.35	457.85	733.04	217.3	1137.6
	Fluctuation Fixation Size # 3 (mm)	217.55	114.76	191.04	132.55	271.82	6.72	661.35
	Mean Fixation Area # 3 (mm^2^)	305.12	105.58	297.99	232.51	372.49	79.62	631.77
	Fluctuation Fixation Area # 3 (mm^2^)	119.97	52.74	112	81.48	151.27	21.43	312.17
Saccadic Features	Mean Saccade Latency (s)	0.55	0.13	0.52	0.45	0.61	0.33	1.13
	Saccade Latency Variability (s)	0.39	0.21	0.34	0.24	0.51	0.07	1.3
	Total Saccadic Path Length in diagonal sequences (deg)	260.2	118.51	225.22	178.92	308.68	67.83	835.45
	Total Diagonal Saccadic Path Length (deg)	144.34	51.15	134.57	110.27	166.87	30.44	447.39
	Median Diagonal Saccadic Velocity (deg/s)	302.31	47.65	302.73	274.3	330.38	137.76	473.56
	Total Saccadic Path length in horizontal sequences (deg)	257.11	121.01	228.37	172.8	303.94	108.18	956.91
	Total Horizontal Saccadic Path Length (deg)	140.23	42.01	134.27	115.26	156.63	51.26	363.26
	Median Horizontal Saccadic Velocity (deg/s)	324.84	47.67	327.06	296.89	358.66	155.56	458.85
	Total Saccadic Path Length in vertical sequences (deg)	284.21	121.7	256.22	203.53	332.64	104.13	909.44
	Total Vertical Saccadic Path Length (deg)	170.77	92.12	148.06	109.65	210.51	33.42	670.77
	Median Vertical Saccadic Velocity (deg/s)	278.82	58.16	285.13	238.4	320.47	130.22	433.75
Blink Features	Blink Rate (blinks per minute)	17.91	12.22	16.11	8.18	25.51	0	60.74
	Mean Blink Duration (ms)	195.33	69.83	184.33	148.35	234.50	0.00	476.00
	Fluctuation Blink Duration (ms)	111.63	77.58	102.91	42.42	169.70	0.00	321.82
Pupillary Features	Mean Pupil Diameter (mm)	4.09	0.78	4.02	3.62	4.56	1.96	6.42
	Fluctuation in Pupil Diameter (mm)	0.28	0.11	0.26	0.21	0.32	0.11	1.01
	Pupil Coefficient of Variation (%)	6.74	1.70	6.41	5.67	7.36	3.20	17.56

**Table 3:** Demographic-Adjusted Normative Regression Models.

	OCAT Features	Intercept β0 [95% CI]	Age β1 [95% CI]	Sex β2 [95% CI]	Education β3 [95% CI]	APOE β4 [95% CI]	RMSE (Original Units)	Adjusted R^2^
Time-related Cognitive Performance Features	Total OCAT Test Time (s)	75.71 [58.13–93.29]	0.045 [−0.13–0.22]	−0.54 [−3.83–2.74]	−0.68 [−1.42–0.051]	−1.81 [−4.71–1.09]	13.8	0.017
	Mean Time # 1 (s)	1.33 [0.89–1.77]	0.0058 * [0.002–0.01]	−0.051 [−0.13–0.032]	−0.0016 [−0.02–0.017]	−0.0049 [−0.08–0.07]	0.35	0.026
	Std. Dev. Time #1 (s) Model: ln(Y)	−1.73 [−2.39–−1.06]	0.011 * [0.004–0.017]	−0.15 * [−0.29–−0.002]	−0.0016 [−0.031–0.027]	−0.047 [−0.17–0.079]	0.24	0.04
	Mean Time # 2 (s)	2.12 [1.61–2.64]	−0.0008 [−0.006–0.004]	0.0069 [−0.089–0.1]	−0.02 * [−0.040–−0.00]	−0.074 [−0.16–0.008]	0.39	0.02
	Std. Dev. Time # 2 (s)	−0.13 [−0.80–0.53]	−0.0018 [−0.008–0.005]	0.023 [−0.12–0.17]	−0.042 * [−0.07–−0.013]	−0.14 * [−0.26–−0.008]	0.27	0.035
	Mean Time # 3 (s)	2.86 [2.2–3.51]	−0.0013 [−0.008–0.005]	−0.0013 [−0.13–0.12]	−0.035 * [−0.06–−0.007]	−0.071 [−0.18–0.04]	0.52	0.025
	Std. Dev. Time # 3 (s)	0.47 [−0.19–1.13]	−0.0052 [−0.012–0.002]	0.029 [−0.1–0.16]	−0.034 * [−0.06–−0.004]	−0.037 [−0.17–0.09]	0.46	0.022
Fixation Features	Mean Fixation Time # 1 (ms)	1116.32 [721.4–1511.3]	2.39 [−1.58–6.36]	−26.53 [−113.4–60.35]	−2.37 [−19.69–14.96]	10.69 [−64.5–85.9]	346.4	0.005
	Fluctuation Fixation Time # 1 (ms) Model: ln(Y)	5.29 [4.81–5.78]	0.0094 * [0.005–0.014]	−0.064 [−0.17–0.042]	−0.0053 [−0.027–0.016]	0.0084 [−0.084–0.1]	166.63	0.044
	Mean Fixation Size # 1 (mm)	416.40 [240.11–592.7]	1.18 [−0.38–2.74]	4.05 [−31.17–39.27]	−0.53 [−8.03–6.97]	1.52 [−28.11–31.14]	137.21	0.005
	Fluctuation Fixation Size # 1 (mm) Model: ln(Y)	4.63 [4.15–5.11]	0.0063 * [0.001–0.011]	−0.03 [−0.14–0.08]	−0.0057 [−0.027–0.015]	−0.02 [−0.11–0.078]	67.95	0.021
	Mean Fixation Area # 1 (mm^2^)	199.93 [92.49–307.36]	1.2 * [0.12–2.28]	9.66 [−13.97–33.3]	0.61 [−4.1–5.33]	−9.31 [−29.77–11.15]	94.23	0.021
	Fluctuation Fixation Area # 1 (mm^2^)	83.39 [28.01–138.76]	0.78 * [0.22–1.33]	6.51 [−5.67–18.69]	−1.37 [−3.79–1.06]	−0.88 [−11.42–9.67]	48.56	0.032
	Mean Fixation Time # 2 (ms)	1566.18 [1107–2025.7]	−2.64 [−6.91–1.64]	69.36 [−26.3–165.05]	−10.27 [−29.27–8.74]	−6.62 [−81.02–67.78]	347.41	0.014
	Fluctuation Fixation Time # 2 (ms) Model: ln(Y)	6.19 [5.68–6.69]	−0.002 [−0.007–0.003]	0.11 * [0.007–0.21]	−0.018 [−0.04–0.003]	0.006 [−0.088–0.1]	157.30	0.020
	Mean Fixation Size # 2 (mm)	518.96 [354.5–683.4]	−0.33 [−1.98–1.32]	39.15 [2.97–75.33]	−1.64 [−8.86–5.58]	−2.34 [−33.7–28.9]	144.27	0.014
	Fluctuation Fixation Size # 2 (mm)	195.15 [123.26–7.03]	−0.24 [−0.96–0.49]	16.34 [0.52–32.15]	−2.52 [−5.68–0.63]	4.52 [−9.17–18.2]	63.05	0.021
	Mean Fixation Area # 2 (mm^2^)	226.07 [127.5–324.7]	0.68 [−0.31–1.67]	15.89 [−5.8–37.57]	−0.49 [−4.8–3.83]	−12.42 [−31.19–6.36]	86.47	0.019
	Fluctuation Fixation Area # 2 (mm^2^)	94.44 [50.12–138.75]	0.34 [−0.1–0.79]	10.17 [0.42–19.9]	−1.24 [−3.18–0.71]	−5.01 [−13.45–3.42]	38.87	0.03
	Mean Fixation Time # 3 (ms)	2109.55 [1583–2635.7]	−3.95 [−9.24–1.33]	59.10 [−56.6–174.8]	−19.26 [−44.34–3.83]	11.55 [−88.6–111.73]	461.48	0.016
	Fluctuation Fixation Time #3 (ms) Model: ln(Y)	6.62 [6.03–7.21]	−0.0043 [−0.01–0.002]	0.06 [−0.074–0.19]	−0.0130 [−0.039–0.013]	−0.0124 [−0.125–0.1]	285.85	0.01
	Mean Fixation Size # 3 (mm)	757.51 [544.9–970.17]	−1.16 [−3.3–0.97]	38.97 [−7.81–85.74]	−5.30 [−14.64–4.03]	4.05 [−36.4–44.54]	186.52	0.014
	Fluctuation Fixation Size # 3 (mm) Model: ln(Y)	5.78 [5.16–6.4]	−0.0063 * [−0.013–−0.0]	0.12 [−0.02–0.25]	−0.011 [−0.038–0.017]	0.04 [−0.078–0.16]	114.92	0.022
	Mean Fixation Area # 3 (mm^2^)	265.39 [142.5–388.26]	0.98 [−0.134–2.1]	9.90 [−17.06–36.86]	−1.27 [−6.81–4.28]	−17.69 [−40.01–4.64]	105	0.019
	Fluctuation Fixation Area # 3 (mm^2^)	123.90 [63.6–184.21]	0.35 [−0.25–0.96]	1.79 [−11.47–15.06]	−1.59 [−4.24–1.06]	−3.5625 [−15.05–7.92]	52.9	0.01
Saccadic Features	Mean Saccade Latency (s) Model: ln(Y)	−0.95 [−1.2–−0.7]	0.0055 * [0.003–0.008]	−0.015 [−0.07–0.04]	−0.0011 [−0.012–0.01]	−0.029 [−0.077–0.018]	0.13	0.048
	Saccade Latency Variability (s)	−1.22 [−1.84–0.6]	0.0019 [−0.004–0.008]	0.017 [−0.12–0.15]	0.0032 [−0.024–0.03]	−0.1 [−0.22–0.014]	0.21	0.011
	Total Saccadic Path Length in diagonal sequences (deg) Model: ln(Y)	5.29 [4.82–5.75]	0.0052 * [0.001–0.01]	−0.0062 [−0.11–0.095]	−0.0079 [−0.028–0.012]	−0.0487 [−0.14–0.04]	117.54	0.021
	Total Diagonal Saccadic Path Length (deg) Model: ln(Y)	4.47 [4.1–4.85]	0.0056 * [0.002–0.009]	−0.0139 [−0.097–0.07]	0.0058 [−0.011–0.022]	−0.0278 [−0.1–0.044]	50.19	0.028
	Median Diagonal Saccadic Velocity (deg/s)	304.30 [251.14–357.5]	−0.55 * [−1.09–−0.018]	−8.88 [−20.58–2.81]	2.17 [−0.17–4.5]	2.64 [−7.48–12.76]	46.63	0.031
	Total Saccadic Path Length in horizontal sequences (deg) Model: ln(Y)	5.28 [4.83–5.74]	0.0032 [−0.001–0.008]	0.019 [−0.082–0.12]	−0.0016 [−0.022–0.019]	−0.035 [−0.12–0.053]	121.15	0.009
	Total Horizontal Saccadic Path Length (deg) Model: ln(Y)	5.03 [4.7–5.36]	0.0 [−0.003–0.003]	0.0084 [−0.065–0.081]	−0.0065 [−0.021–0.008]	−0.043 [−0.11–0.02]	42.03	0.008
	Median Horizontal Saccadic Velocity (deg/s)	363.50 [309.8–417.22]	−0.4145 [−0.93–0.1]	−4.60 [−17.79–8.59]	−0.62 [−2.9–1.65]	0.26 [−10.07–10.6]	47.19	0.01
	Total Saccadic Path Length in vertical sequences (deg) Model: ln(Y)	5.49 [5.07–5.91]	0.0042 [0–0.008]	0.012 [−0.081–0.11]	−0.01 [−0.03–0.008]	−0.051 [−0.081–0.11]	121.4	0.021
	Total Vertical Saccadic Path Length (deg) Model: ln(Y)	4.86 [4.27–5.45]	0.002 [−0.004–0.008]	−0.053 [−0.18–0.076]	0.0031 [−0.023–0.029]	−0.035 [−0.15–0.077]	91.77	0.004
	Median Vertical Saccadic Velocity (deg/s)	274.12 [208.93–339.3]	−0.022 [−0.68–0.63]	−16.35 * [−30.69–−2.01]	0.77 [−2.09–3.63]	−3.27 [−15.68–9.15]	57.12	0.017
Blink Features	Blink Rate (blinks per minute)	8.52 [−5.2–22.23]	−0.35 [−0.021–0.26]	−4.61 * [−7.62–−1.59]	0.2 [−0.4–0.8]	−0.35 [−2.96–2.26]	12.03	0.032
	Mean Blink Duration (ms)	254.97 [176.43–333.5]	−0.4 [−1.19–0.39]	−12.06 [−29.34–5.22]	−1.23 [−4.68–2.21]	−22.48 * [−37.44–−7.53]	68.89	0.036
	Fluctuation Blink Duration (ms)	144.48 [57.69–31.28]	0.12 [−0.75–0.99]	−20.21 * [−39.31–−1.12]	−1.63 [−5.44–2.18]	−19.85 * [−36.38–−3.33]	76.13	0.033
Pupillary Features	Mean Pupil Diameter (mm)	5.60 [4.77–6.43]	−0.0271 * [−0.035–−0.02]	−0.16 [−0.35–0.019]	0.0204 [−0.016–0.057]	−0.0877 [−0.25–0.07]	0.73	0.13
	Fluctuation in Pupil Diameter (mm) Model: ln(Y)	−0.73 [−1.09–−0.37]	−0.0087 * [−0.01–−0.005]	−0.02 [−0.099–0.06]	−0.0005 [−0.016–0.015]	−0.048 [−0.12–0.021]	0.1	0.068
	Pupil Coefficient of Variation (%) Model: ln(Y)	2.13 [1.87–2.39]	−0.0022 [−0.005–0.00]	0.024 [−0.033–0.081]	−0.0059 [−0.017–0.005]	−0.026 [−0.075–0.024]	1.69	0.014

* Significant values *p* < 0.05.

**Table 4:** Diagnostic assessment of the normative regression models.

OCAT Features	Residual Skewness	Kurtosis	Breusch-Pagan *p*
Total OCAT Test Time (s)	0.47	3.24	0.0009 *
Mean Time # 1 (s)	0.58	3.29	0.004 *
Std. Dev. Time #1 (s)	0.13	2.67	0.48
Mean Time # 2 (s)	0.78	4.09	0.016 *
Std. Dev. Time # 2 (s)	0.012	2.61	0.22
Mean Time # 3 (s)	0.49	3.14	0.007 *
Std. Dev. Time # 3 (s)	0.01	2.54	0.008 *
Mean Saccade Latency (s)	0.63	3.19	0.22
Saccade Latency Variability (s)	−0.13	2.71	0.46
Mean Fixation Time # 1 (ms)	0.42	3.49	0.052
Fluctuation Fixation Time # 1 (ms)	0.19	2.92	0.49
Mean Fixation Size # 1 (mm)	0.42	2.94	0.01 *
Fluctuation Fixation Size # 1 (mm)	0.11	2.65	0.29
Mean Fixation Area # 1 (mm^2^)	0.34	2.87	0.31
Fluctuation Fixation Area # 1 (mm^2^)	0.89	3.81	0.07
Mean Fixation Time # 2 (s)	0.41	3.61	0.02 *
Fluctuation Fixation Time # 2 (ms)	−0.084	2.8	0.68
Mean Fixation Size # 2 (mm)	0.63	3.53	0.62
Fluctuation Fixation Size # 2 (mm)	0.95	3.59	0.65
Mean Fixation Area # 2 (mm^2^)	0.38	2.95	0.76
Fluctuation Fixation Area # 2 (mm^2^)	0.85	3.89	0.18
Mean Fixation Time # 3 (s)	0.46	3.12	0.06
Fluctuation Fixation Time # 3 (ms)	−0.07	3.59	0.23
Mean Fixation Size # 3 (mm)	0.46	2.82	0.09
Fluctuation Fixation Size # 3 (mm)	0.19	3.16	0.13
Mean Fixation Area # 3 (mm^2^)	0.38	2.96	0.03 *
Fluctuation Fixation Area # 3 (mm^2^)	0.8	3.75	0.92
Total Saccadic Path Length in diagonal sequences (deg)	0.39	3.15	0.98
Total Diagonal Saccadic Path Length (deg)	−0.14	4.61	0.75
Median Diagonal Saccadic Velocity (deg/s)	−0.03	3.45	0.23
Total Saccadic Path Length in horizontal sequences (deg)	0.59	3.23	0.95
Total Horizontal Saccadic Path Length (deg)	−0.06	3.64	0.23
Median Horizontal Saccadic Velocity (deg/s)	−0.39	3.54	0.04 *
Total Saccadic Path Length in vertical sequences (deg)	0.53	3.2	0.55
Total Vertical Saccadic Path Length (deg)	−0.15	3.11	0.35
Median Vertical Saccadic Velocity (deg/s)	−0.12	2.55	0.46
Blink Rate (blinks per minute)	0.74	3.09	0.14
Mean Blink Duration (ms)	0.74	4.88	0.06
Fluctuation Blink Duration (ms)	0.48	2.41	0.69
Mean Pupil Diameter (mm)	0.28	3.26	0.96
Fluctuation in Pupil Diameter (mm)	0.4	3.98	0.54
Pupil Coefficient of Variation (%)	0.56	4.19	0.48

* Significant values *p* < 0.05.

**Table 5:** Illustrative demographic-adjusted reference values for the OCAT-derived fixation-related feature related to Fluctuation Fixation Time #1.

Age, Years	Sex	Education, Years	APOE Status	Predicted Fluctuation Fixation Time #1	Lower 2.5th Percentile	Upper 97.5th Percentile	95% Reference Interval
50	Male	16	Non-carrier	273.9	118.6	632.9	118.6–632.9
50	Male	16	ɛ4-carrier	276.2	119.6	638.3	119.6–638.3
50	Female	16	Non-carrier	292.1	126.4	675	126.4–675.0
50	Female	16	ɛ4-carrier	294.6	127.5	680.7	127.5–680.7
60	Male	16	Non-carrier	300.8	130.2	695.1	130.2–695.1
60	Male	16	ɛ4-carrier	303.4	131.3	701	131.3–701.0
60	Female	16	Non-carrier	320.8	138.9	741.3	138.9–741.3
60	Female	16	ɛ4-carrier	323.6	140	747.6	140.0–747.6
70	Male	16	Non-carrier	330.4	143	763.4	143.0–763.4
70	Male	16	ɛ4-carrier	333.2	144.2	769.8	144.2–769.8
70	Female	16	Non-carrier	352.4	152.5	814.1	152.5–814.1
70	Female	16	ɛ4-carrier	355.3	153.8	821	153.8–821.0
80	Male	16	Non-carrier	362.8	157	838.4	157.0–838.4
80	Male	16	ɛ4-carrier	365.9	158.4	845.5	158.4–845.5
80	Female	16	Non-carrier	387	167.5	894.1	167.5–894.1
80	Female	16	ɛ4-carrier	390.3	168.9	901.7	168.9–901.7

**Table 6:** Illustrative demographic-adjusted reference values for the OCAT-derived saccade-related feature related to Mean Saccade Latency.

Age, Years	Sex	Education, Years	APOE Status	Predicted Saccade Latency	Lower 2.5th Percentile	Upper 97.5th Percentile	95% Reference Interval
50	Male	16	Non-carrier	0.49	0.32	0.76	0.32–0.76
50	Male	16	ɛ4-carrier	0.48	0.31	0.74	0.31–0.74
50	Female	16	Non-carrier	0.5	0.32	0.77	0.32–0.77
50	Female	16	ɛ4-carrier	0.49	0.31	0.75	0.31–0.75
60	Male	16	Non-carrier	0.52	0.34	0.8	0.34–0.8
60	Male	16	ɛ4-carrier	0.5	0.33	0.78	0.33–0.78
60	Female	16	Non-carrier	0.53	0.34	0.81	0.34–0.81
60	Female	16	ɛ4-carrier	0.51	0.33	0.79	0.33–0.79
70	Male	16	Non-carrier	0.55	0.36	0.85	0.36–0.85
70	Male	16	ɛ4-carrier	0.53	0.35	0.82	0.35–0.82
70	Female	16	Non-carrier	0.56	0.36	0.86	0.36–0.86
70	Female	16	ɛ4-carrier	0.54	0.35	0.83	0.35–0.83
80	Male	16	Non-carrier	0.58	0.38	0.89	0.38–0.89
80	Male	16	ɛ4-carrier	0.56	0.37	0.87	0.37–0.87
80	Female	16	Non-carrier	0.59	0.38	0.91	0.38–0.91
80	Female	16	ɛ4-carrier	0.57	0.37	0.88	0.37–0.88

## Data Availability

The raw data supporting the conclusions of this article will be made available by the authors on request.

## Abbreviations

The following abbreviations are used in this manuscript:

OCAT	Oculo-Cognitive Addition Test
AVLT	Auditory Verbal Learning Task
DRS	Dementia Rating Scale
APOE ε4	apolipoprotein E Type 4
RMSE	Root Mean Squared Error
